# Computational repurposing of approved drugs targeting KRAS G12D and EGFR for colorectal cancer therapy

**DOI:** 10.1371/journal.pone.0338123

**Published:** 2026-01-28

**Authors:** Walaa Ibraheem, Fatima Alsheikh, Abdulrahim A. Alzain, Bhoomendra A. Bhongade, Laila Ejaz, Hamnah Baig, Robert C. Robinson, Mohamed El-Tanani

**Affiliations:** 1 RAK College of Pharmacy, RAK Medical & Health Science University, RAK, UAE; 2 School of Biomolecular Science and Engineering (BSE), Vidyasirimedhi Institute of Science and Technology (VISTEC), Rayong, Thailand; 3 Division of Microbiology, Department of Pharmaceutics, Faculty of Pharmacy, University of Gezira, Gezira, Sudan; 4 Department of Pharmaceutical Chemistry, Faculty of Pharmacy, University of Gezira, Gezira, Sudan; 5 RAK College of Medicine, RAK Medical & Health Science University, RAK, UAE; Jamia Millia Islamia Central University: Jamia Millia Islamia, INDIA

## Abstract

**Background/objectives:**

Colorectal cancer is characterized by various oncogenic mutations, with the KRAS ^G12D^ mutation being the most prevalent. The development of MRTX1133 has revitalized the KRAS direct targeting. However, colorectal cancer demonstrated intrinsic resistance to MRTX1133, primarily due to the feedback activation of the EGFR pathway. Combining KRAS ^G12D^ and EGFR inhibition has demonstrated improved treatment efficacy, highlighting the potential of dual-targeting approaches in colorectal cancer therapy. This study employs CADD tools to identify approved drugs capable of dual targeting KRAS ^G12D^ and EGFR.

**Methods:**

A library of 3,591 approved drugs was screened against KRAS ^G12D^ using high-throughput virtual screening (HTVs), standard precision (SP) and extra precision (XP) Glide docking modules. The top-ranking compounds were then docked into the EGFR binding pocket using XP mode, and docking scores were calculated. Further refinement of binding affinities was performed using Molecular Mechanics with Generalized Born and Surface Area Solvation (MM-GBSA) and Molecular Dynamics (MD) simulations.

**Results:**

A total of 25 drugs showed better affinity to KRAS ^G12D^ than MRTX1133 (−9.18 kcal/mol). Among them, six drugs: Reproterol, Macimorelin, Nebivolol, Nadolol, Antrafenine, and Carteolol, displayed docking scores between −7.186 and −8.864 kcal/mol against EGFR, compared to the reference ligand Erlotinib (−9.669 kcal/mol). Subsequent MM-GBSA and MD identified Carteolol, an FDA-approved beta blocker, as the most promising candidate, showing stable and reliable binding with KRAS ^G12D^ and relatively stable interactions with EGFR.

**Conclusions:**

This *in silico* study predicts Carteolol as a potential dual-targeting therapeutic agent, requiring biochemical and cellular validation before clinical relevance can be established.

## 1. Introduction

Kirsten rat sarcoma viral oncogene (KRAS) plays a significant role in tumor initiation and progression in a broad spectrum of human cancers. They account for >80% of all RAS mutations and are implicated in 20% of all human malignancies. The incidence of KRAS mutations is particularly high in colorectal cancer (CRC), occurring in 35–40% of CRC cases. Especially, the KRAS ^G12D^ mutation is a more dominant variant found in 12% of CRC cases and is significantly associated with a high risk of metastasis and a poor prognosis [1–3].

KRAS is stimulated by extracellular growth signals mediated by the epidermal growth factor receptor (EGFR) family. This growth signaling allows KRAS to shuttle between binding with guanosine-5′-triphosphate (GTP) in an ‘on’ state and with guanosine-5′-diphosphate (GDP) in an ‘off’ state. Mutations in KRAS alter the dynamics of this cycle, resulting in hyperactive KRAS, which constitutively activates three major pathways downstream: the mitogen-activated protein (MAP) kinase pathway, the PI3K/AKT/mTOR (PAM) pathway, and the tumor invasion and metastasis-inducing protein 1 (TIAM1–RAC) and RAS-related protein (RAL) pathways. This uncontrolled signaling results in increased proliferation, decreased apoptosis, and increased angiogenesis, contributing to oncogenesis. Therefore, directly targeting mutant KRAS could serve as an effective therapeutic strategy [1,2,4,5].

Targeting of KRAS has been considered quite challenging. Preliminary studies of the KRAS proteins revealed that they lack a hydrophobic binding cavity other than the GTP/GDP binding pocket. However, GTP is naturally abundant within cells and designing effective nucleotide**-**competitive inhibitors presents a significant challenge. Furthermore, targeting the KRAS upstream or downstream effectors has either failed to produce desired results or led to significant adverse effects. A breakthrough in the field occurred in 2013 when advancements in understanding KRAS biology led to the identification of the first KRAS ^G12C^ inhibitor [2].

The identification of a new allosteric site within KRAS ^G12C^ has led to the development of covalent inhibitors targeting the reactive Cys12 in the KRAS^G12C^ mutant through structure-based design. However, the G12D mutation is the most common mutational event in KRAS, and only a small percentage of patients experience positive outcomes from G12C-targeted therapy. Nevertheless, the development of specific KRAS ^G12C^ inhibitors revealed that the allosteric site in all KRAS proteins is a valid target for designing selective KRAS ^G12D^ inhibitors. In 2021, a selective noncovalent KRAS ^G12D^ inhibitor named MRTX1133 was developed, and preclinical studies indicate that it has the potential to revolutionize the approach to treating KRAS ^G12D^ -driven cancer, potentially reshaping the current therapeutic strategies [3,6–9]. Despite MRTX1133 being an effective inhibitor of KRAS ^G12D^ and demonstrating a cytotoxic effect on KRAS ^G12D^ -mutated cancer cells, a recent report concluded that KRAS ^G12D^ -mutated colorectal cancer cells exhibit a reduced sensitivity **to** MRTX1133, resulting in a weaker therapeutic response. This study revealed that feedback activation of the EGFR/wild-type RAS pathway reduces the effectiveness of KRAS ^G12D^ inhibitor therapy in CRC. Notably, wild isoforms of RAS, rather than oncogenic KRAS, trigger downstream signaling of the activated EGFR, resulting in a rebound reactivation and reduced MRTX1133 efficacy. Fortunately, the inhibition of this pathway with kinase inhibitors such as erlotinib blocked EGFR/wild-type RAS signaling and sensitized cells to MRTX1133 [10].

Drug repurposing emphasizes on identifying new therapeutic uses for existing approved and marketed drugs. Drugs that have already undergone clinical trials are proven safe with well-known pharmacokinetic, pharmacodynamic, and toxicity profiles, allowing researchers to explore their broader therapeutic potential. Compared to the 12–13 years and 2–3 billion USD needed to bring a new drug from bench to market, repurposing affords a faster and more affordable alternative, saving both cost and time by approximately 30% [11]. Moreover, the integration of computational approaches during the initial stages of drug discovery has proven to be invaluable. These approaches are now frequently employed at different phases of the drug development process, such as hit recognition and lead optimization. Virtual screening enables the narrowing of a vast array of compounds for experimental testing and the identification of promising drug candidates. Molecular docking is widely utilized in virtual screening, especially when the 3D structure of a protein is known. Moreover, molecular dynamics (MD) has emerged as a computational tool for exploring the flexibility and dynamics of drug‒target interactions, revolutionizing the field of computer-aided drug design (CADD) [12–14]. Together, drug repurposing and advanced computational methods expedite the development pipeline, offering a cost-effective and time-saving pathway to identify effective therapeutic solutions [15].

This study utilizes computational methods to explore the repurposing potential of existing drugs to concurrently target KRAS ^G12D^ and EGFR. This dual-targeting strategy is intended to circumvent resistance and enhance the therapeutic efficacy of KRAS ^G12D^ -directed treatments.

## 2. Materials and methods

### 2.1. Computational resources

Maestro v 12.8 of the Schrodinger suite was used in this study.

### 2.2. Preparation of proteins and ligands

The three-dimensional (3D) structures of KRAS ^G12D^ (PDB ID: 7RPZ) and EGFR tyrosine kinase (PDB ID: 1M17) were retrieved from the Protein Data Bank (https://www.rcsb.org/structure). The two proteins were then prepared via the protein preparation wizard tool Maestro, which refines and optimizes the protein structures. The proteins were subsequently minimized via the OPLS3e force field [16,17].

The binding cavities of the proteins were defined around the co-crystallised ligands MRTX1133 and Erlotinib bound to the protein via the Receptor Grid Generation tool of Maestro. The coordinates of the ligands with the protein were used to create a 3D grid with precise dimensions, representing the receptor’s active area [18].

A library of 3,591 approved drugs was obtained from the ChEMBL library (https://www.ebi.ac.uk/chembl/). During this process, Epik was employed to generate the most probable protonation and tautomeric states at physiological pH (7.0 ± 2.0). Subsequently, all compounds were energy minimized using the OPLS3e force field to generate low-energy conformers to ensure that the compound structures were acceptable for further computational work [19].

### 2.3. Molecular docking and MM-GBSA calculations

The prepared library served as an input for a multistage virtual screening to identify potential dual inhibitors for KRAS ^G12D^ and EGFR. Initially, the library was screened against KRAS G12D, and ligands were shortlisted based on their binding strength to the receptor, represented by the Glide score (G score). This process involved sequential docking steps via the Glide module. High-throughput virtual screening (HTVS) is used for quick and initial screening, followed by standard perception (SP) and extra precision (XP), which provides the highest accuracy. Default parameters were employed for the docking process, and the results were analyzed based on the G-scores [20–22].

**Fig 1:**
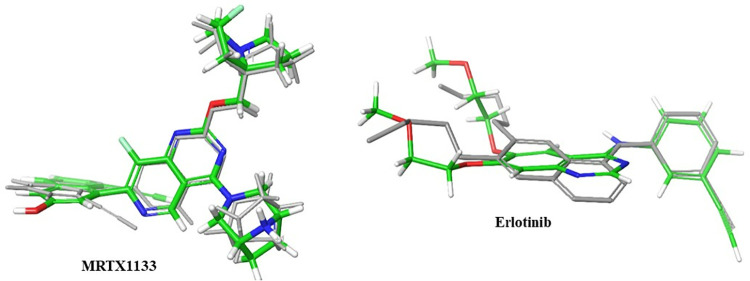
The 3D structures of the redocked MRTX1133 and Erlotinib(Grey)superimposed on the co-crystalized structures (Green).

Compounds that achieved high docking scores were further docked into the binding pocket of EGFR, and their docking score were calculated. The docking protocol was validated by redocking the native co-crystallized inhibitors into the prepared active sites of KRAS ^G12D^ and EGFR. In both cases, the re-docked poses closely reproduced the binding orientations observed in the co-crystallized structures, with root mean square deviation (RMSD) values of 2.06 Å and 1.09 Å, respectively (Fig 1).

The docking poses of the shortlisted compounds were then used to calculate their MM-GBSA binding free energy via the Prime tool of Maestro. MM-GBSA is a rescoring method that demonstrates a strong correlation between predicted and observed activity [23–25]. The binding free energy was calculated via the following equation [24]:


ΔG bind = G complex − (G protein + G ligand)

Where:

∆G bind = binding free energy

G complex = free energy of the complex

G protein = free energy of the target protein

G ligand = free energy of the ligand.

The calculations applied the VSGB implicit solvation model and the OPLS3e force field, as integrated into the Schrödinger Prime module. The VSGB model approximates solvation effects without explicitly including solvent molecules, and the calculations were performed in an implicit solvent environment, not in vacuum. Additionally, entropy contributions were omitted, which is a common simplification in MM-GBSA workflows to reduce computational cost while maintaining reliable relative binding energy estimates [26].

### 2.4. Molecular dynamics (MD) simulations

The Glide XP docking output files were input for MD simulations conducted using the GROMACS 2024.4 software package. Ligand topologies were generated using the SwissParam server, while protein parameters were generated using the OPLS-AA/L force field and TIP3P water model within GROMACS [27]. A triclinic simulation box was created around the protein-ligand complexes using the gmx editconf module. For EGFR, the box was set to an orthorhombic shape, and the protein-drug complex was positioned at the center of the 11.4 x 8.6 x 7.2 nm box. The KRAS ^G12D^ protein-drug complex was positioned at the center of a cubic box with a 6 nm distance. Water molecules were added to fully solvate the systems, along with Na ions to neutralize the system, and pairs of Na and Cl ions were added to achieve a 0.1 M salt concentration. Following box preparation, the system underwent energy minimization using the steepest descent algorithm. Subsequently, NVT (constant number of particles, volume, and temperature) and NPT (constant number of particles, pressure, and temperature) equilibration runs were performed for 100 ps to achieve a stable system at the desired temperature and pressure. The stability of the complexes was assessed using root means square deviation (RMSD), root mean square fluctuation (RMSF), and radius of gyration (Rg).

### 2.5. In silico ADME prediction

The physicochemical characteristics of the highest-scoring ligands were analysed using the pkCSM web server, accessible at https://biosig.unimelb.edu.au/pkcsm/. This tool facilitates the prediction of ADME parameters to evaluate the drug-likeness of the compounds.

## 3. Results and discussion

The emergence of targeted therapies, such as the selective inhibitor MRTX1133, has revolutionized the treatment of KRAS ^G12D^-mutated cancers [7]. While this compound has demonstrated cytotoxic effects on KRAS ^G12D^-mutated cancer cells, recent reports revealed that CRC cells harbouring this mutation often display diminished responsiveness to MRTX1133 [28]. The reduced efficacy has been attributed to feedback activation of the EGFR/wild-type RAS pathway. However, the inhibition of activated EGFR has been shown to suppress this compensatory signalling, thereby increasing sensitivity to MRTX1133 [10]. Given these challenges, our study aims to employ CADD to assess the potential of repurposing approved drugs to inhibit both the KRAS ^G12D^ and EGFR proteins. By employing CADD, we can efficiently screen a library of approved drugs to identify candidates that may simultaneously target both the KRAS ^G12D^ and the EGFR. This approach enhances the therapeutic efficacy and overcomes the limitations posed by feedback mechanisms in CRC treatment.

The active sites for KRAS and EGFR were determined using the bound ligands to both proteins. The active site of KRAS (PDB ID: 7RPZ) bound to MRTX1133 was validated using the SiteMap of the Schrodinger suite which showed site-1 residues were consistent with the interacting residues of MRTX1133 bound to KRAS (S1 Table, supplementary file)

**Fig 2:**
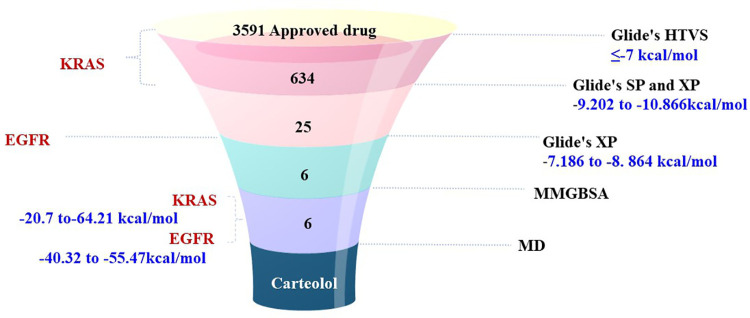
Summary of the study workflow.

As represented in Fig 2, the workflow involved two rounds of docking. In the 1st round, a library of 3,591 approved drugs was screened against KRAS ^G12D^. Consecutive docking steps employing the Glide module were implemented to filter the most promising candidate from this library and to analyze the nature of interactions between the target and ligands. Regardless of its limitation in accounting for the flexibility of receptors, docking remains a valid tool for providing preliminary insights. Preliminary screening via Glide’s HTVS mode revealed that 634 compounds presented docking scores of −7 kcal/mol or lower. These compounds were then subjected to SP docking, and the top 100 compounds with SP scores < −8.2 kcal/mol were selected for further refinement. Extra Precision (XP) docking was subsequently performed on this subset. To prioritize compounds with superior predicted binding affinities, we applied a selection criterion based on outperforming the reference ligand MRTX1133, which had an XP docking score of −9.18 kcal/mol. Accordingly, compounds with lower XP scores were shortlisted. This resulted in 25 top-ranking compounds with XP scores ranging from −9.202 to −10.866 kcal/mol, all demonstrating stronger predicted binding than the reference. The top-ranked were subsequently selected for a second round of XP docking against the EGFR. The EGFR docking results indicated that six approved drugs, namely Reproterol, Macimorelin, Nebivolol, Nadolol, Antrafenine and Carteolol, achieved docking scores ranging between −7.186 to −8. 864 kcal/mol. In comparison, the reference ligand Erlotinib exhibited a docking score of −9.669 kcal/mol.

The strength of the binding affinity for the top six drugs was evaluated by calculating the binding free energy. The obtained dGbind values ranged from-20.7 to-64.21 kcal/mol for KRAS ^G12D^ and −40.32 to –55.47 kcal/mol for EGFR (Table 1). The reference ligands MRTX1133 and Erlotinib exhibited an MM-GBSA value of −27.15 and −65.58 kcal/mol, respectively (Table 1). These calculations were performed without including entropy contributions, a common simplification in MM-GBSA workflows aimed at reducing computational cost. However, this omission represents a notable limitation, especially when interpreting absolute binding energies, which may lack accuracy in the absence of entropic terms. Despite this, relative binding energy comparisons remain robust and continue to provide valuable insights into ligand ranking and binding strength. less accurate without entropic terms. Relative binding energy comparisons remain reliable under this approach and continue to offer meaningful insights into ligand ranking and binding strength.

**Table 1:** The docking and MM/GBSA result for the shortlisted drugs and reference ligands.

Drug name	Chembl ID	XP Gscore KRAS^G12D^	XP Gscore EGFR	MMGBSA KRAS^G12D^	MMGBSA EGFR
**Reproterol**	CHEMBL1095607	−7.935	−8.864	−37.93	−40.81
**Nebivolol**	CHEMBL434394	−10.006	−8.123	−29	−46.32
**Nadolol**	CHEMBL521606	−10.989	−7.816	−64.21	−49.65
**Macimorelin**	CHEMBL278623	−9.49	−7.638	−15.74	−49.2
**Carteolol**	CHEMBL839	−9.372	−7.185	−57.95	−49.26
**Antrafenine**	CHEMBL345524	−9.101	−7.856	−20.7	−55.47
**Erlotinib**			−9.669		−65.58
**MRTX1133**		−9.18		−27.15	

Notably, our primary objective is not to identify candidates with superior EGFR binding compared to Erlotinib, but rather to identify compounds that exhibit strong affinity for KRAS ^G12D^ while also modulating EGFR. This dual-targeting strategy aims to address resistance mechanisms often encountered in colorectal cancer. Therefore, these compounds were taken forward for detailed structural and binding interaction analysis.

Interestingly, among the shortlisted compounds, three beta-adrenoceptor blockers (Nebivolol, Nadolol and Carteolol) and one short-acting agonist (Reproterol) were identified. Notably, the six shortlisted compounds share common structural features, including a protonated primary (as in the case of Macimorelin) or secondary amine group, a bicyclic nucleus, and alkyl substituents. These structural elements contribute to the formation of an extensive hydrophobic interface, which has the potential to serve as an effective framework for developing KRAS ^G12D^ inhibitors [29].

**Fig 3:**
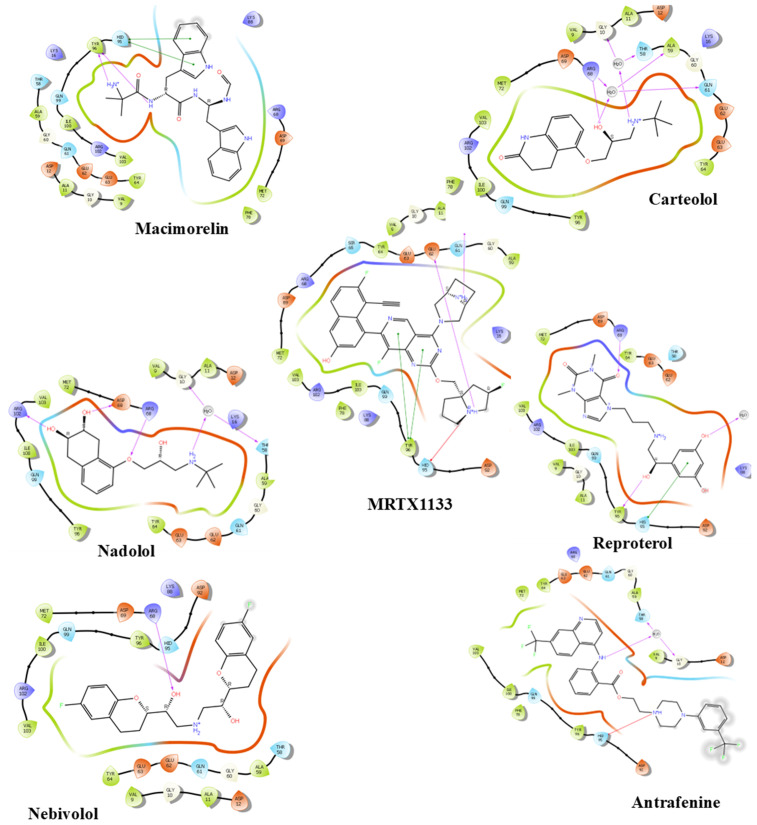
The two-dimensional (2D) interactions of the shortlisted drugs and MRTX1133 with KRAS ^G^12^D^ (PDB ID: 7RPZ). The hydrogen bond interactions with residues are represented in a purple arrow, while a red-blue arrow represents the salt bridge, hydrophobic interactions are represented with green residues, polar interactions with faint blue residues, positive interactions with dark blue residues and negative interactions with red residues.

It has been reported that the key amino acids contributing to the stability of KRAS ^G12D^, in descending order of significance, are: Asp69, His95, Met72, Gln99, Arg68, Tyr96, Tyr64, Gly60, Asp12, and Val9 [29]. As shown in Fig 2 and Table 2, all the shortlisted compounds, except for Macimorelin and Antrafenine, exhibited a shared interaction with Arg68. Macimorelin, however, formed a hydrogen bond with Tyr96 and a pi-pi stacking interaction with His95, mirroring the interactions observed in Reproterol. In reproterol, though, the interaction with His95 was through a hydrogen bond. Antrafenine, Carteolol and Nadolol establish a structured hydrogen bond network. This network enables the ligands to effectively connect the lipophilic and polar regions of the switch II pocket. This network is established through a water bridge, involving the conserved water molecules within the binding site. In this interaction, the hydroxyl group of Thr58 and the carbonyl oxygen of Gly10 function as hydrogen acceptors, while the amine groups of the ligands serve as hydrogen donors. Nadolol also forms hydrogen bonds with Asp69 and Arg102. Prior research suggests that inhibitors forming such networks are associated with a stronger inhibitory effect [9]. Conversely, MRTX1133 is stabilized in the binding pocket through hydrogen bonds with Asp12 and Glu62, as well as pi‒pi and pi-cation interactions with Tyr96 and His95, respectively (Fig 3 and Table 2).

**Table 2:** The intermolecular interactions of the shortlisted drugs and MRTX1133 with KRAS ^G12D.^

Drug name	H-bond	Water bridge	π-cation	π-π stacking	Hydrophobic interaction	Others
**Reproterol**	H_2_O, Arg68, Hid 95, Tyr96				Val9. Ala11, Val14, Pro34, Ala59, Phe78, Met72, Tyr64, Tyr96, Ile100, Val103	Polar: Ser17, Thr35, Thr58, Hid95, Gln99
						Charged negative: Asp69, Glu62, Glu63, Asp92
						Charged positive: Arg68, Lys88Arg102
						Gly10
**Nebivolol**	Arg68				Val9. Ala11, Val14, Ala59, Phe78, Met72, Tyr64, Tyr96, Ile100, Val103	Polar: Thr58, Gln61 Hid95, Gln99
						Charged negative: Asp12, Asp69, Glu62, Glu63, Asp92
						Charged positive: Arg68, Lys88 Arg102
						Gly10, Gly60
**Nadolol**	H_2_O Arg68, Asp69, Arg102	Gly10, Thr58			Val9. Ala11, Ala59, Met72, Tyr64, Tyr96, Ile100, Val103	Polar: Thr58, Gln61, Gln99
						Charged negative: Asp12, Asp69, Glu62, Glu63.
						Charged positive Lys16 Arg68Arg102
						Gly10, Gly60
**Macimorelin**	Tyr96			Hid95	Val9. Ala11, Ala59, Phe78, Met72, Tyr64, Tyr96, Ile100, Val103	Polar: Thr58, Gln61 Hid95, Gln99
						Charged negative: Asp12, Asp69, Glu62, Glu63.
						Charged positive Lys16, Lys88Arg102
						Gly10, Gly60
**Carteolol**	Arg68	Gly10, Thr58, Ala59, Gln61, Arg68			Val9. Ala11, Ala59, Phe78, Met72, Tyr64, Tyr96, Ile100, Val103	Polar: Thr58, Gln61, Gln99
						Charged negative: Asp12, Glu62, Glu63, Asp69.
						Charged positive Lys16, Arg68, Arg102
						Gly10, Gly60
**Antrafenine**		Gly10, Thr58	Hid95		Val9. Ala11, Met72, Tyr64, Tyr96, Ile100, Val103	Polar: Thr58, Hid95, Gln99
						Charged negative: Glu62, Glu63, Asp69, Asp92
						Charged positive Arg68, Lys88, Arg102
						Gly10
**MRTX1133**	Asp12, Glu62		Hid95	Tyr96	Val9. Ala11, Ala59, Phe78, Met72, Tyr64, Tyr96, Ile100, Val103	Polar: Ser65, Gln61, Hid95, Gln99
						Charged negative: Asp12, Asp69, Glu62, Glu63
						Charged positive: Lys16, Arg68, Arg102

**Fig 4:**
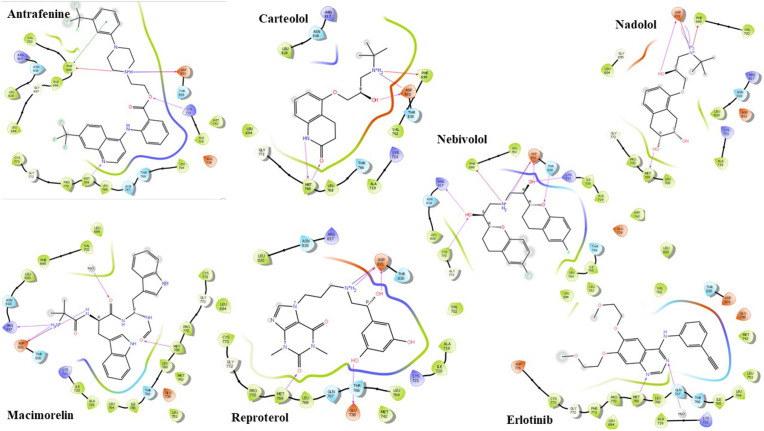
The two-dimensional (2D) interactions of the shortlisted drugs and Erlotinib with EGFR (PDB ID: 1M17). The hydrogen bond interactions with residues are represented in a purple arrow, while a red-blue arrow represents the salt bridge, hydrophobic interactions are represented with green residues, polar interactions with faint blue residues, positive interactions with dark blue residues and negative interactions with red residues.

Furthermore, the structural attributes of the selected compounds facilitate the formation of a robust hydrogen bond network within EGFR’s ATP binding pocket. A sustained hydrogen bond with Met769 was noted, along with an ionic interaction between the cationic amino groups of these compounds and the carboxylic acid side chain of Asp831. Previous research has reported that the interaction with Asp831 can enhance EGFR inhibition by 50-fold [30]. It is worth mentioning that nebivolol, while not interacting with Met769, did interact with Cys773. This interaction was pivotal in the creation of irreversible EGFR inhibitors, which exhibited significant anti-tumor activity. Effects in both in vitro and in vivo investigations, all while maintaining minimal cytotoxicity [9]. Moreover, Antrafenine, Carteolol, Nadolol, and Nebivolol exhibited additional interactions with Phe699. Nebivolol and Antrafenine also showed interactions with Lys721. Nadolol and Macimorelin interacted with a water molecule in the binding site, while Macimorelin and Nebivolol interacted with Arg817. Reproterol and Nebivolol uniquely interacted with Glu738 and Thr830, respectively. Erlotinib formed hydrogen bonds with Met769 and water molecules in the EGFR binding site (Fig 4 and Table 3).

**Table 3:** The intermolecular interactions of the shortlisted drugs and Erlotinib with EGFR.

Drug name	H-bond	Salt bridge	π-π stacking	π-Cation	Hydrophobic interaction	Others
**Reproterol**	Asp831 Glu738, Met769	Asp831			Leu694, Val702, Ala719, Ile720, Met742, Leu764, Leu768, Met769, Pro770, Cys773 Leu820.	Polar: Thr766, Gln767, Asn818, Thr830
						Charged negative: Glu738, Asp831
						Charged positive: Lys721, Arg817
						Gly772
**Nebivolol**	Lys721, Cys773, Arg817, Asp831, Thr830	Asp831		Phe699	Leu694, Phe699, Val702, Ile720, Met742, Leu764, Ile765, Cys773 Leu820.	Polar: Thr766, Asn818, Thr830
						Charged negative: Glu738, Asp831
						Charged positive: Lys721, Arg817
						Gly772
**Nadolol**	H2O Met 769, Asp831	Asp831		Phe699	Leu694, Phe699, Val702, Ala719, Leu768, Met769, Leu820.	Polar: Thr766, Gln767, Thr830
						Charged negative: Asp831, Asp813
						Charged positive: Lys721, Arg817
						Gly695
**Macimorelin**	H2O Met 769, Asp831 Arg817	Asp831			Leu694, Phe699, Val702, Ala719, Met742, Leu753, Leu768, Met769, Cys773 Leu753, Leu764, Ile765 Leu820.	Polar: Thr766, Asn818, Thr830
						Charged negative: Asp776, Glu738, Asp831.
						Charged positive: Lys721, Arg817
						Gly772
**Carteolol**	Met 769, Asp831	Asp831	Phe699		Leu694, Phe699, Val702, Ala719, Leu768, Met769, Leu820.	Polar: Thr766, Thr830
						Charged negative: Asp831
						Charged positive: Lys721
						Gly772
**Antrafenine**	Met 769, Lys721	Asp831	Phe699	Asp831 Phe699	Leu694, Ala698, Phe699, Val702, Ala719, Met742, Leu753, Leu768, Met769, Pro770, Phe771, Cys773 Leu753, Leu764, Ile765 Leu820, Phe832.	Polar: Thr766, Gln767, Asn818, Thr830
						Charged negative: Asp831, Glu738
						Charged positive: Lys721, Arg817
						Glys697, Gly772
**Erlotinib**		Water, Met769			Leu694Val702, Ala719, Met742, Pro770, Cys773, Phe771Leu768, Met769, Leu753, Leu764, Ile765 Leu820,	Polar: Gln767, Thr766
						Charged negative: Asp831, Glu738, Asp776
						Charged positive: Lys721
						Gly772

**Fig 5:**
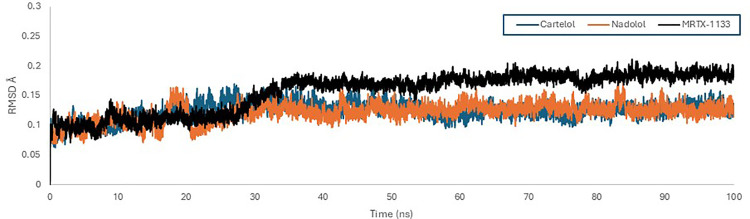
Root mean square deviation (RMSD) graphical record of KRAS ^G^12^D^ (PDB ID: 7RPZ) with Carteolol (Blue), Nadolol (orange)and MRTX1133 (Black) over a 100 ns simulation time using GROMACS software.

**Fig 6:**
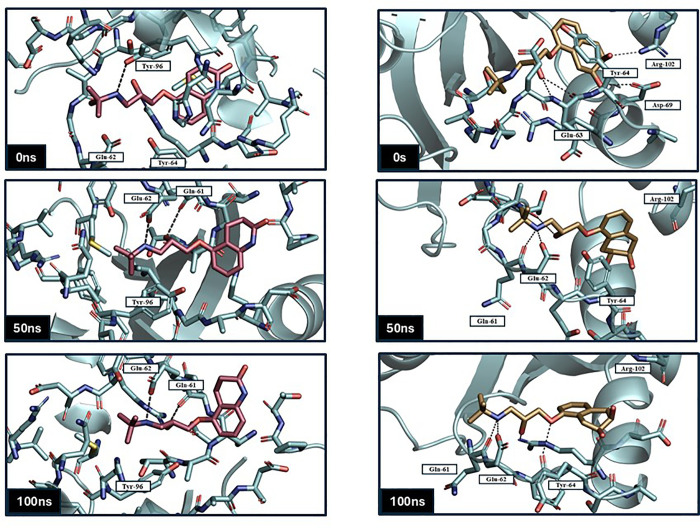
Representative snapshots of Carteolol (Red) and Nadolol (Sand)within KRAS ^G^12^D^ binding pocket at 0 ns, 50 ns, and 100 ns during the molecular dynamics simulation over a 100 ns simulation time using GROMACS software.

The flexibility of the receptor is crucial for reliably predicting drug binding affinity, thermodynamic properties, and kinetic behavior. Integrating docking studies with MD simulations can therefore improve the screening process and facilitate the identification of novel lead compounds [31]. Nadolol and Carteolol were identified as promising candidates, demonstrating the highest docking scores with KRAS ^G12D^ and acceptable scores with EGFR. The compounds were then analyzed using 100 ns molecular dynamics (MD) simulations to assess their binding stability. Key parameters analyzed included the root mean square deviation (RMSD), the root mean square fluctuation (RMSF), and the radius of gyration of the ligand. The RMSD is used to quantify the average movement of a selection of atoms in a specific

frame compared to a reference frame. Analyzing RMSD provides valuable insights into a protein’s structural conformation throughout the simulation, where lower values and fewer deviations signify higher stability. The RMSD values were calculated and displayed on a graph, where the x-axis represents simulation time ranging from 0 to 100 ns, and the y-axis shows RMSD values in nanometers (nm). The RMSD statistics were presented in S2 and S3 Table in the supplementary files. The RMSD plot for the KRAS ^G12D^ protein revealed that all three ligands, Nadolol, Carteolol, and MRTX1133 maintained a stable interaction with the KRAS ^G12D^ protein throughout the simulation, as evidenced by the low RMSD values. The curves of Carteolol (blue) and Nadolol (orange) are closely aligned, displaying a stable pattern with negligible fluctuations, ranging between 0.06 nm and 0.16 nm and an average RMSD of 0.124 nm. In contrast, the reference ligand (black) exhibited slightly higher RMSD values ranging between 0.059 nm and 0.21 nm, and an average RMSD of 0.156 nm, indicating greater fluctuations compared to Carteolol and Nadolol, suggesting that MRTX1133 leads to a less stable conformation of the KRAS ^G12D^ protein (Fig 5). To further illustrate the conformational evolution of the ligand throughout the simulation, snapshots at 0 ns, 50 ns, and 100 ns were represented to capture the initial, intermediate, and final binding pose (Fig 6). The visual progression highlights the dynamic nature of ligand–receptor interactions within the binding pocket. Initially, carteolol was stabilized through its interaction with Tyr96. As the simulation progressed, this interaction was replaced by new contacts with Gln61 and Glu62, which ultimately stabilized the ligand in its final binding conformation. Similarly, nadolol exhibited a shift in its binding profile: it was first stabilized by Asp69, Glu63, and Arg102, followed by a transition to Glu62, and eventually settled into a conformation supported by interactions with both Glu62 and Tyr64.

**Fig 7:**
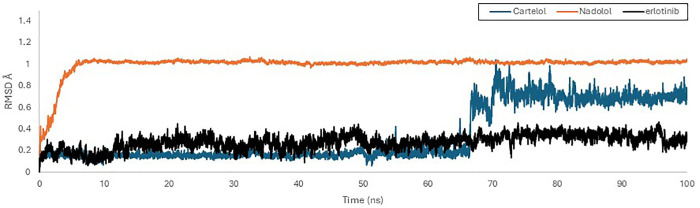
Root mean square deviation (RMSD) graphical record of EGFR (PDB ID: 1M17) with Carteolol (Blue), Nadolol (orange) and Erlotinib (Black) over a 100 ns simulation time using GROMACS software.

In Fig 7, the RMSD analysis of the EGFR-ligand complexes highlights distinct stability patterns among Erlotinib, Carteolol, and Nadolol. The EGFR with Erlotinib in black displayed a stable pattern with minimal fluctuations, maintaining an average RMSD of 0.34 nm. In contrast, Carteolol in blue exhibited an initial equilibrium state around 0.2 nm for the first 68 ns, followed by fluctuations lasting for 8 ns, after which the system restabilized at approximately 0.7 nm until the end of the simulation. This suggests Carteolol experiences some instability and conformational changes within the EGFR binding pocket.

**Fig 8:**
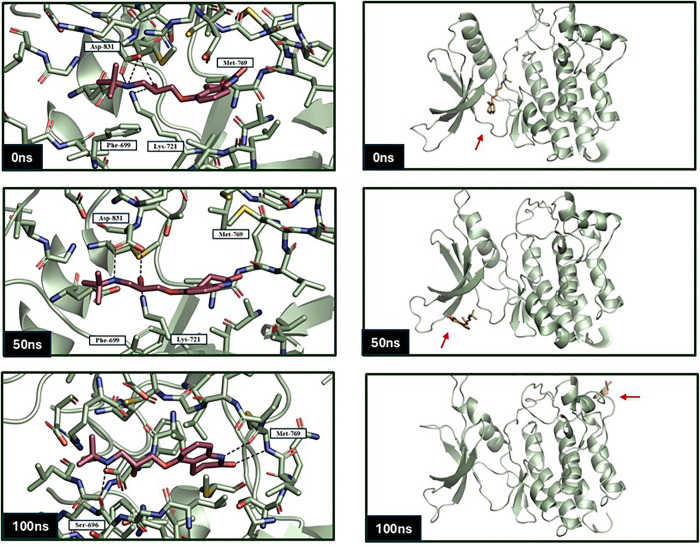
Representative snapshots of Carteolol (Red) and Nadolol (Sand) within the EGFR binding pocket at 0 ns, 50 ns, and 100 ns during the molecular dynamics simulation in EGFR over a 100 ns simulation time using GROMACS software.

As shown in Fig 8, Carteolol, initially stabilized through hydrogen bonds with ASP 831 and LYS 721, maintaining low RMSD for the first 65 ns. Following this phase, the ligand undergoes a structural shift, increasing RMSD. However, the ligand subsequently establishes new interactions with MET 769 and LEU 694, leading to re-stabilization within the binding pocket. This dynamic behavior indicates the ligand can adapt and optimize its binding interactions over time. On the other hand, Nadolol (orange) stabilized within the first 5 ns, but maintained a higher RMSD of 1 nm, which is significantly greater than the other two ligands. This suggests greater conformational variability and possibly weaker binding interactions with the EGFR pocket (Fig 7). Consistent with this, the snapshots in Fig 8 reveal notable positional shifts of nadolol within the binding site over time, reflecting its dynamic and less stable engagement with key residues.

Further analysis of MD trajectories using the Visual Molecular Dynamics (VMD) tool verified that Erlotinib and Carteolol remained within the simulation box, supporting their continued interaction with the target protein (S1 Video). Overall, Erlotinib exhibits the most favorable binding stability, followed by Carteolol, which demonstrates dynamic interaction characteristics, whereas Nadolol’s elevated RMSD suggests less stable binding behavior.

**Fig 9:**
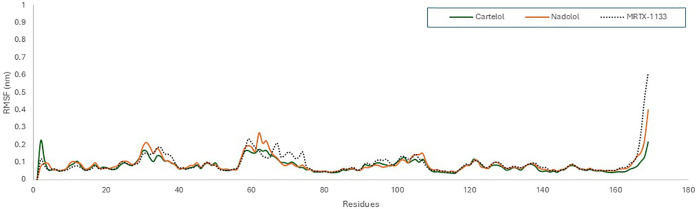
The root means square fluctuation RMSF profile of KRAS ^G^12^D^ (PDB ID: 7RPZ) with Carteolol (green), Nadolol (orange)and MRTX1133 (Black) over 100 ns simulation time using GROMACs software.

**Fig 10:**
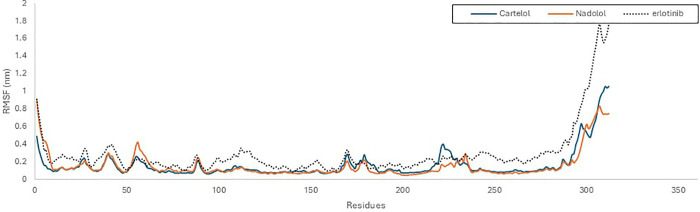
The root means square fluctuation RMSF profile of EGFR (PDB ID: 1M17) with Carteolol (green), Nadolol (orange)and Erlotinib (Black) over a 100 ns simulation time using GROMACs software.

To evaluate the stability of amino acid residues, we conducted RMSF analysis; a statistical tool that quantifies the extent of residue movement throughout a simulation. This analysis helps identify protein regions exhibiting significant fluctuations. The RMSF plot for the KRAS ^G12D^ and EGFR, each bound to Carteolol, Nadolol, and reference ligands, indicated that most residues displayed only slight fluctuations, with RMSF values remaining below 0.3 nm for KRAS ^G12D^ and 0.4 nm for EGFR. It is worth noting that the regions showing higher RMSF values were remote from the binding cavity, reflecting a well-preserved structure in the KRAS ^G12D^ and EGFR when bound to three ligands (Figs 9 and 10). Despite its higher RMSD, Nadolol’s lower RMSF suggests that while the overall structure undergoes significant conformational changes, certain regions of the protein remain stable. This indicates that nadolol may stabilize specific areas of the protein, even as the complex undergoes broader structural changes throughout the simulation.

**Fig 11:**
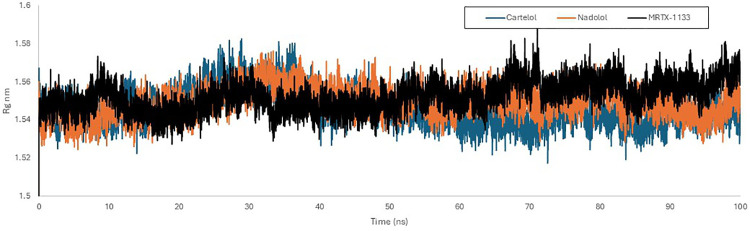
Radius of gyration graph of KRAS ^G^12^D^ (PDB ID: 7RPZ) with Carteolol (Blue), Nadolol (orange) and MRTX1133 (Black) over 100 ns simulation time using GROMACs software.

The radius of gyration (Rg) was utilized as an additional parameter to validate the stability of the protein-ligand complexes as indicated by the RMSD and RMSF findings. Rg measures the distance between the center of mass and the rotational axis of the ligand-protein complex. It represents a measure of the protein structure’s compactness and stability throughout the simulation. Lower Rg values indicate greater stability and compactness of the complex, whereas higher values imply reduced stability and compactness. As depicted in Fig 11 the radius of gyration plot for the KRAS ^G12D^ protein illustrates that the three ligands—Carteolol (blue), Nadolol (orange), and MRTX1133 (black)—generally exhibit Rg values ranging from 1.53 to 1.58 nm throughout the simulation, indicating that all complexes maintain a comparable level of compactness. This finding aligned with the results obtained from RMSD and RMSF analyses.

**Fig 12:**
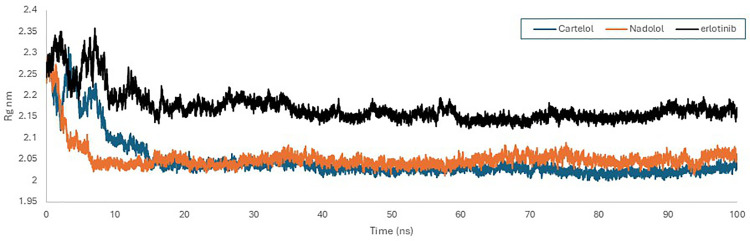
Radius of gyration graph EGFR (PDB ID: 1M17) with Carteolol (Blue), Nadolol (orange)and MRTX1133 (Black) over a 100 ns simulation time using GROMACs software.

The Radius of Gyration (Rg) plot for the EGFR reveals that both Nadolol and Carteolol induce a more compact conformation compared to Erlotinib, as reflected by their lower Rg values (Fig 12). Although Erlotinib has a lower RMSD than Nadolol and Carteolol, this does not contradict the Rg analysis. The lower RMSD indicates that Erlotinib maintains stable binding interaction, but it may not achieve the same level of compactness as Nadolol and Carteolol.

The ADMET prediction results from the pkCSM server provide important information about the pharmacokinetics and toxicity of the selected compounds (Table 4). Water solubility values (log mol/L) ranged from −2.704 to −3.622, showing different solubility levels; Nadolol and Carteolol were less soluble than Erlotinib. Blood-brain barrier permeability (log BB) values indicated that most compounds have limited ability to cross into the brain, with values between −0.682 and −1.62, except Erlotinib, which had a positive value (0.427), suggesting it can penetrate the barrier more easily. This may affect its activity or side effects in the central nervous system. In terms of distribution, Nadolol did not significantly interact with cytochrome P450 enzymes CYP2D6 and CYP3A4, while Carteolol acted as both a substrate and inhibitor of these enzymes, which may influence its metabolism and potential drug interactions.Metabolism predictions showed differences among compounds regarding cytochrome P450 enzyme interactions. MRTX1133 was not a substrate for CYP2D6 or CYP3A4 but inhibited both enzymes, which could affect metabolic pathways. Erlotinib inhibited CYP1A2 but was neither a substrate nor inhibitor of the other tested enzymes. Regarding excretion, none of the compounds were substrates for renal OCT2, affecting their elimination routes. Toxicity predictions indicated Nadolol and Carteolol tested negative for AMES toxicity. All compounds showed hepatotoxicity except Erlotinib.

**Table 4:** The ADMET prediction of the top two drugs and the references.

Compound	Absorption	Distribution	Metabolism						Excretion	Toxicity	
			CYP								
			2D6	3A4	2D6	3A4	1A2	2C9			
	Water solubility	Blood-brain barrier permeability	Substrate		Inhibitor				Renal OCT2 substrate	AMES toxicity	Hepatotoxicity
	Numeric (log mol/L)	Numeric (log BB)	Categorical (Yes/No)						Categorical (Yes/No)	Categorical (Yes/No)	
**Nadolol**	-2.704	-0.682	No	No	No	No	No	No	No	No	Yes
**Carteolol**	-3.622	-0.835	Yes	Yes	No	No	No	No	No	No	Yes
**MRTX1133**	-3.443	-1.62	No	No	No	Yes	Yes	No	No	No	Yes
**Erlotinib**	-2.892	0.427	No	No	No	No	Yes	No	No	Yes	No

The findings overall suggest that Carteolol demonstrate notable dual-targeting potential, achieving docking scores of −9.372 kcal/mol for KRAS ^G12D^ and −7.185 kcal/mol for EGFR. Although both interactions were favorable, it displayed stronger binding affinity toward KRAS ^G12D^. This was further supported by MM-GBSA binding free energy values, with Carteolol exhibiting −57.95 kcal/mol for KRAS ^G12D^ compared to −49.26 kcal/mol for EGFR, reinforcing its preferential binding to the KRAS. Molecular dynamics simulations substantiated these findings by revealing stable and compact interactions with both proteins throughout the simulation period, especially in the KRAS ^G12D^ complex, suggesting robust conformational stability. ADMET prediction showed acceptable properties. The Canvas mean Tanimoto similarity scores using the Canvas’s Schrodinger suite indicate very low structural similarity between Nadolol and Carteolol compared to the reference compounds MRTX1133 and Erlotinib. Specifically, Nadolol showed a similarity score of 0.022, while Carteolol had a slightly higher score of 0.036. These values suggest that both Nadolol and Carteolol share minimal chemical features with MRTX1133 and Erlotinib.

Altogether, these results highlight Carteolol’s potential as a promising small-molecule agent with preferential affinity for KRAS ^G12D^ and complementary modulation of EGFR, aligning with the study’s objective to address resistance mechanisms in colorectal cancer via a dual-targeting strategy. Carteolol is a long-acting, non-selective beta-adrenoceptor antagonist used primarily for the treatment of hypertension. It is also an effective therapy for arrhythmias, angina, and glaucoma. Beyond its cardiovascular applications, beta blockers are gaining recognition in oncology, with both preclinical and clinical studies indicating potential therapeutic benefits outside their conventional use, demonstrating novel mechanisms of action not linked to β-adrenergic receptor blockade. This supports the exploration of new mechanisms that go beyond their traditional roles. Furthermore, β-blockers are suggested to function as immunomodulators. Studies have shown that adrenergic stimulation can enhance cancer hallmarks, such as modulating apoptosis and increasing angiogenesis, effects that beta blockers can counteract. However, the safety profiles of repurposed β-blockers depend on specific conditions and patient characteristics, highlighting the need for further research to optimize their application in non-cardiovascular contexts. Despite these challenges, their potential for treating chronic diseases like cancer positions β-blockers as promising candidates for therapeutic innovation across a broader range of medical fields [11,32,33].

## 4. Conclusions

This study has employed Insilico tools to identify potential dual-targeting agents for KRAS ^G12D^ and EGFR in colorectal cancer. By screening a library of 3,591 approved drugs through molecular docking, binding free energy calculation and subsequent molecular dynamics simulations, Carteolol emerged as a promising candidate, demonstrating strong binding affinity and stability with both targets. While these computational tools are limited in their capacity to validate biological efficacy fully, they offer a valuable starting point for hypothesis generation and hit identification. Experimental validation via in vitro and in vivo assays is essential to confirm these findings and advance the therapeutic potential of Carteolol in colorectal cancer.

## Supporting information

S1. Video 100 ns molecular dynamics simulation of KRAS ^G12D^ (PDB ID: 7RPZ) with Carteolol (using GROMACs software. (ZIP)

S1 Table Prediction of the active site of KRAS using SiteMap. (DOCX)

S2 Table Statistics of RMSD of the top two drugs and the references bond to KRAS during 100 ns MD simulation. (DOCX)

S3 Table Statistics of RMSD of the top two drugs and the references bond to EGFR during 100 ns MD simulation. (DOCX)
