# Supplementary material for: Computational repurposing of approved drugs targeting KRAS G12D and EGFR for colorectal cancer therapy
Source: PLoS One. 2026 Jan 28;21(1):e0338123. doi: 10.1371/journal.pone.0338123 (PMC12851494; doi:10.1371/journal.pone.0338123)
Supplement: S1 Table — (DOCX) [file pone.0338123.s002.docx]

Table S1: Prediction of the active site of KRAS using SiteMap:

| Title | size | Dscore | SiteScore | volume-1 | residues |
| --- | --- | --- | --- | --- | --- |
| site_1 | 152 | 1.056 | 1.093 | 273.714 | Chain  A: 9,10,11,12,13,16,34,58,59,60,61,62,63,64,68,69,72,78,92,95,96,99,100,102,103 |
| site_2 | 104 | 0.81 | 1.033 | 221.578 | Chain  A: 10,11,12,13,14,15,16,17,18,28,29,30,32,33,34,36,57,58,59,60,116,117,119,120,145,146,147 |
| site_3 | 55 | 0.716 | 0.799 | 116.62 | Chain  A: 97,101,107,108,109,110,111,137,138,139,140,162,165,166,169 |
| site_4 | 34 | 0.792 | 0.782 | 68.6 | Chain A: 3,5,6,7,39,54,56,71,74,75 |
