# Supplementary material for: Computational repurposing of approved drugs targeting KRAS G12D and EGFR for colorectal cancer therapy
Source: PLoS One. 2026 Jan 28;21(1):e0338123. doi: 10.1371/journal.pone.0338123 (PMC12851494; doi:10.1371/journal.pone.0338123)
Supplement: S2 Table — (DOCX) [file pone.0338123.s003.docx]

Table S2: Statistics of RMSD of the top two drugs and the reference bond to KRAS during 100 ns MD simulation

|  | Mean | Standard Deviation | Minimum | Median | Maximum |
| --- | --- | --- | --- | --- | --- |
| Cartelol | 0.34583 | 0.25768 | 5.473E-4 | 0.17927 | 1.04038 |
| Nadolol | 1.00035 | 0.10087 | 5.02E-4 | 1.01948 | 1.06906 |
| erlotinib | 0.28064 | 0.06925 | 5.446E-4 | 0.28531 | 0.46613 |
