# Supplementary material for: Computational repurposing of approved drugs targeting KRAS G12D and EGFR for colorectal cancer therapy
Source: PLoS One. 2026 Jan 28;21(1):e0338123. doi: 10.1371/journal.pone.0338123 (PMC12851494; doi:10.1371/journal.pone.0338123)
Supplement: S3 Table — (DOCX) [file pone.0338123.s004.docx]

Table S3: Statistics of RMSD of the top two drugs and the reference bond to EGFR during 100 ns MD simulation

|  | Mean | Standard Deviation | Minimum | Median | Maximum |
| --- | --- | --- | --- | --- | --- |
| Cartelol | 0.1242 | 0.01502 | 4.863E-4 | 0.12584 | 0.1752 |
| Nadolol | 0.12159 | 0.0158 | 5.065E-4 | 0.12362 | 0.17221 |
| MRTX-1133 | 0.15683 | 0.03277 | 5.06E-4 | 0.17132 | 0.21157 |
